# Sex-specific associations between subcortical morphometry in childhood and adult alcohol consumption: A 17-year follow-up study

**DOI:** 10.1016/j.nicl.2021.102771

**Published:** 2021-07-26

**Authors:** Catherine Mankiw, Ethan T. Whitman, Erin Torres, François Lalonde, Liv S. Clasen, Jonathan D. Blumenthal, M. Mallar Chakravarty, Armin Raznahan

**Affiliations:** a Section on Developmental Neurogenomics, Human Genetics Branch, National Institute of Mental Health, Bethesda, MD, USA; b Computational Brain Anatomy (CoBrA) Laboratory, Cerebral Imaging Centre, Douglas Mental Health University Institute, Montreal, Quebec, Canada

**Keywords:** Alcohol, Amygdala, Hippocampus, Longitudinal predictors, Sex differences, Subcortical anatomy

## Abstract

Men and women tend to differ in the age of first alcohol consumption, transition into disordered drinking, and the prevalence of alcohol use disorder. Here, we use a unique longitudinal dataset to test for potentially predispositonal sex-biases in brain organization prior to initial alcohol exposure. Our study combines measures of subcortical morphometry gathered in alcohol naive individuals during childhood (mean age: 9.43 years, SD = 2.06) with self-report measures of alcohol use in the same individuals an average of 17 years later (N = 81, 46 males, 35 females). We observe that pediatric amygdala and hippocampus volume both show sex-biased relationships with adult drinking. Specifically, females show a stronger association between subcortical volumetric reductions in childhood and peak drinking in adulthood as compared to males. Detailed analysis of subcortical shape localizes these effects to the rostro-medial hippocampus and basolateral amygdala subnuclei. In contrast, we did not observe sex-specific associations between striatal anatomy and peak alcohol consumption. These results are consistent with a model in which organization of the amygdala and hippocampus in childhood is more relevant for subsequent patterns of peak alcohol use in females as compared to males. Differential neuroanatomical precursors of alcohol use in males and females could provide a potential developmental basis for well recognized sex-differences in alcohol use behaviors.. Thus, our findings not only indicate that brain correlates of human alcohol consumption are manifest long before alcohol initiation, but that some of these correlates are not equivalent between males and females.

## Highlights

• 17-Year follow up linking pediatric brain anatomy with adult drinking.
• Female-specific relationships between subcortical anatomy and adult drinking.
• Effects localize to subregions of the amygdala and hippocampus.

## 1 Introduction

Early detection of individuals at-risk for developing alcohol use disorders is a clinical and public health priority, and recent evidence suggests that epidemiologic and biologic predictors of alcohol use are present in early childhood, years before initiation (Bjork et al., 2017). A wide range of behavioral, biological, and socioeconomic factors predispose individuals to disordered alcohol use (Collins, 2016, Enoch, 2013, Guo et al., 2001). One such factor is biological sex: males and females have been reported to differ on several facets of alcohol use disorder, including prevalence (Center for Behavioral Health Statistics and Quality, 2018), initial exposure to alcohol (Greenfield et al., 2010, Keyes et al., 2010), course of illness (Becker et al., 2017, McHugh et al., 2018), and psychiatric comorbidity (Conway et al., 2006, Khan et al., 2013). For example, females consistently tend to initiate drinking later than males (Greenfield et al., 2010, Keyes et al., 2010) and a recent population-based study has demonstrated that males tend to show a faster progression to disordered drinking compared to females (Keyes et al., 2010). Furthermore, from a developmental perspective, there is some evidence that males and females also differ in aspects of impulsivity and executive function (Klenberg et al., 2001, Yuan et al., 2008) that also predict initiation and increases in drinking (Kwako et al., 2019, Tschorn et al., 2021). These observations could potentially reflect aspects of sex-biased brain organization before exposure to alcohol that differentially relate to alcohol use behaviors in later life.

Sex-differences in epidemiological and clinical aspects of alcohol use disorders are accompanied by evidence for sex-biased neuroimaging correlates of alcohol use. For example, alcohol use has been associated with greater reductions in cortical thickness in males compared to females (Morris et al., 2019), drinking has shown inverse associations with cortical volumes in males and females (Kvamme et al., 2016, Sawyer et al., 2017, Squeglia et al., 2012), and drinking appears to have sex-specific effects on regional white matter volumes (Ruiz et al., 2013). However, the cross-sectional nature of these studies makes it unclear if sex-biased imaging findings are precursors or consequences of sex-differences in alcohol use. Furthermore, while there is emerging cross-sectional evidence for sex-biased subcortical anatomy changes in alcohol use disorder (Grace et al., 2021), neuroimaging studies of sex-differences in the field of alcohol research have largely been dominated by studies of the cortex, rather than subcortical structures (Kvamme et al., 2016, Morris et al., 2019, Sawyer et al., 2017, Squeglia et al., 2012). This imbalance leads to a notable gap in our understanding because (i) several lines of evidence implicate subcortical structures in the neurobiology of alcohol use disorders (in particular amygdala (Laakso et al., 2000, Sawyer et al., 2017), hippocampus (Agartz et al., 1999, Laakso et al., 2000, Schneider et al., 2001, Sullivan et al., 2010), and striatum (Sawyer et al., 2017, Szabo et al., 2004)), (ii) subcortical structures are implicated in many other sex-biased mental disorders (Dougherty et al., 2016, Ho et al., 2019, Nolan et al., 2020), and (iii) subcortical systems are known to undergo sex-biased development en route to and during life-stages of initial exposure to alcohol (Dennison et al., 2013, Goddings et al., 2014, Raznahan et al., 2014, Wierenga et al., 2018). Taken together, the above observations raise the question of whether variation in subcortical anatomy in earlier development - before initiation of drinking - may differentially relate to alcohol use in later life between males and females.

Here, to begin addressing these gaps in knowledge, we relate pediatric morphometry of the hippocampus, amygdala, and striatum to adult alcohol use, using a unique longitudinal neuroimaging cohort, with visits that span an unprecedented average of 17 years between baseline imaging and follow-up measures of alcohol use in adulthood. We use an advanced multi-atlas segmentation algorithm to estimate size and shape of these subcortical structures during childhood (Chakravarty et al., 2013). This automated algorithm has been shown to yield highly reliable segmentations by incorporating information from a large library of dataset-specific atlases (Chakravarty et al., 2013, Raznahan et al., 2014) - offering advantages in application to developing populations (Raznahan et al., 2014). We finally test for associations between subcortical anatomy and alcohol consumption in adulthood and use multivariate linear regression to ask whether any of these associations are modulated by sex.

## 2 Methods

### 2.1 Participants

Our study includes 81 participants (males = 46, females = 35) with available measures of pediatric brain anatomy and adult alcohol use (Clinical trial reg. no. NCT00001246, clinicaltrials.gov; NIH Annual Report Number, ZIAMH002794; (Giedd et al., 2015)). All participants completed a magnetic resonance imaging (MRI) scan in childhood (age 5–12 years, female mean age at scan = 9.32 years [SD = 2.12], male mean age at scan = 9.60 years [SD = 2.00]) and a follow-up self-reported alcohol use questionnaire in adulthood (age 18–35 years, female mean age at follow up = 26.5 years [SD = 3.98], male mean age at follow up = 26.6 years [SD = 4.75]), with a 17-year average time-lapse between baseline and follow up (time-lapse SD = 4.51, time-lapse range: 7.0–24.2 years). Male and female participants did not differ in age at scan (t = -0.60, p = 0.55), age at follow-up (t = -0.09, p = 0.93), or socioeconomic status (SES; t = 0.38, p = 0.70). All participants resided in the United States. Exclusion criteria in childhood included: (i) use of psychiatric medication, enrollment in special education services, history of mental health treatment, or diagnosis of a neurological disorder at baseline, (ii) occurrence of a trauma affecting the nervous system or onset of psychotic disorder between baseline and follow-up, and (iii) parent report of any alcohol consumption by the participant prior to baseline scanning. This protocol was approved by the Institutional Review Board of the National Institute of Mental Health and all participants provided written consent or assent, as appropriate.

### 2.2 Socioeconomic and behavioral measures

Socioeconomic status (SES) was estimated during the first visit using the two-factor Hollingshead measure based on parental education and occupation (Hollingshead and Redlich, 1958). This measure refers to SES of the family at the time of scanning. Adult alcohol use was measured using a self-report questionnaire developed by Molina and Pelham (Molina and Pelham, 2003), to estimate drinks consumed per month during the period of greatest use. This questionnaire asks participants to consider the period of greatest alcohol use in their life and to report their age during that period, the duration of this period, and the daily amount of standard drinks (e.g. one can of beer, glass of wine, or drink of liquor) consumed during this period.

### 2.3 Neuroimaging

MRI scans were completed between 1991 and 2008 using a 1.5 T General Electric Signa scanner at the National Institutes of Health Clinical Center in Bethesda, MD. All T-1 weighted images were gathered on the same axial acquisition protocol with 1.5 mm in-plane resolution and 2.0 mm slice thickness. We used a 3D spoiled-gradient recalled-echo sequence with 5 ms echo time, 24 ms repetition time, 45 degree flip angle, 256 × 192 acquisition matrix, 1 excitation, and 24 cm field of view. Scans were only included with ranks “good” (1) or “fair” (2) according to Blumenthal et al. (Blumenthal et al., 2002). This score was not significantly associated with participant age (t = −1.40, p = 0.18). Thus, all scans included lacked visible marked motion artifacts prior to preprocessing. Included scans also passed visual inspection of subcortical segmentation labels according to Multiple Automatically Generated Templates Brain Segmentation Algorithm (MAGeT-Brain) outputs (see below) (Raznahan et al., 2014). Total brain tissue volume (TBV - total gray + total white matter volume) was estimated using FreeSurfer version 5.3 (Fischl, 2012).

### 2.4 Subcortical segmentation

Measures of size and shape for the amygdala, hippocampus, and striatum (comprising the caudate and putamen) were automatically generated from all pediatric brain scans using the well-validated multi-atlas segmentation algorithm, MAGeT-Brain (Chakravarty et al., 2013). This algorithm customized previously described atlases generated from high-resolution T-1 and T-2 weighted images (Chakravarty et al., 2013, Pipitone et al., 2014, Winterburn et al., 2013) to 21 subjects randomly selected from the NIH Human Brain Development in Health Study (Giedd et al., 2015). This library of atlases was then used as templates to which all other scans are registered for segmentation. This process generated 105 study-specific segmentations of subcortical volumes for each scan (5 original atlases × 21 templates), and final segmentation used the label that most often occurred at a given location. This algorithm has high reliability with previous manual tracing definitions (Pipitone et al., 2014). All scans underwent detailed quality control to identify gross segmentation errors.

MAGeT-Brain uses a marching cubes algorithm to create surface-based visualizations subsequently smoothed by the AMIRA software package in a group-specific atlas of the original 5 atlas images (Voineskos et al., 2015). Nonlinear portions were averaged across 21 input templates resulting in 105 possible representations per subject, which were combined by estimating the median coordinate representation at each point. One third of the adjacent triangle’s surface area was assigned to each vertex. Surface assignments were summed to produce the estimated surface area value at each vertex, and these values were blurred using a diffusion-smoothing kernel of 5 mm. Bilateral subcortical volumes, as well as vertex-level estimates of surface area (n vertices: 2367 amygdala, 2878 hippocampus, 12,628 striatum) for each structure were related to adult alcohol use by the following multivariate linear regression (shown here with surface area as an example dependent variable):

$$\text{Surface Area}=\beta_{0}+\beta_{1}Drinks+\beta_{2}Scan\;Age+\beta_{3}Sex+\beta_{4}\left(\mathrm{Sex}\times Drinks\right)$$

where “Drinks” is a continuous variable for drinks per month at period of peak drinking, and Sex is a binary variable with Female as the reference level. Age was centered at mean age so that the main effect of Sex reflects male–female differences in anatomical measures at the mean age in our sample. This modelling approach enables detection of main and sex-modified associations between pediatric subcortical anatomy and peak alcohol use in adulthood. Vertex-wise analyses of subcortical surface area were corrected for multiple comparisons using false discovery rate (FDR) correction.

### 2.5 Sensitivity analyses

Observation of the distribution of reported drinks per month at peak drinking revealed three individuals with unusually high drinks per month (>150 drinks, approximately the 96th percentile). In order to test whether these individuals were driving the overall effects, multivariate regression analyses were completed both with and without those individuals’ data. Results were similar in both cases (Fig. 2A**)**.

To test for robustness of subcortical findings to control for total brain volume variation, we also conducted separate sensitivity analyses while including total brain volume (TBV: total gray matter volume + total white matter volume) as a covariate. In order to test that our findings were robust to control for SES, we also conducted analyses using SES as a covariate. Finally, to test for lateralized effects we calculated asymmetry indices for each subcortical structure and tested for significant sex-biased shifts in these indices as a function of peak drinking.

## 3 Results

### 3.1 Participant characteristics

**Fig. 1:**
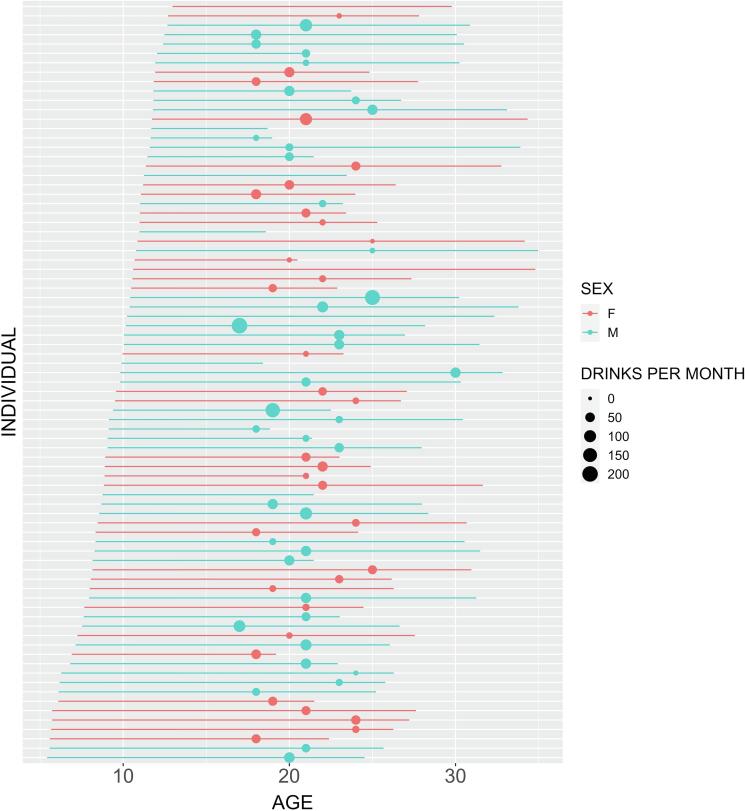
**Visual representation of participants’ study enrollment and peak drinking.** Color encodes sex (red = female / blue = male). Line segments span baseline and follow-up visits. Points along the lines represent the time point of peak drinking, with the size of these points representing the average drinks per month. (For interpretation of the references to color in this figure legend, the reader is referred to the web version of this article.)

Participant age at scan and measures of SES at baseline are summarized in Table 1. The timelines of participants’ baseline visit, follow up visit, and peak drinking are represented in Fig. 1**.**

**Table 1:** Participant Characteristics at Baseline.

Characteristic	Females	Males
Sample Size	35	46
Age at Scan [Mean (S.D.)]	9.32 (2.12)	9.60 (2.00)
SES [Mean (S.D.)]	37.29 (18.13)	35.74 (17.97)

**Fig. 2:**
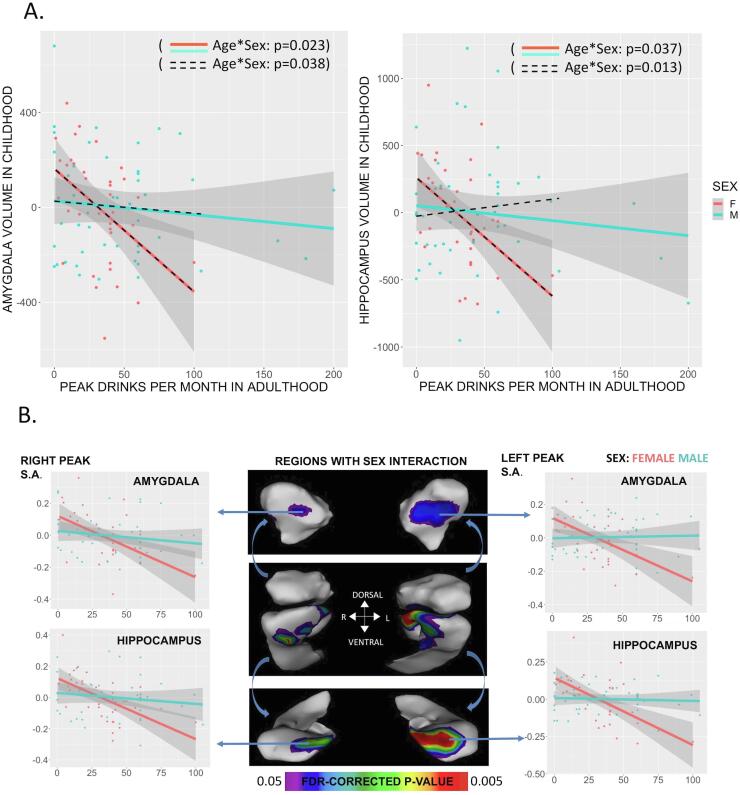
**Sex-specific relationships between amygdalohippocampal anatomy in childhood and alcohol consumption in adulthood. (A)** Size of the amygdala and hippocampus in childhood (age-and-sex-regressed total bilateral volume) show a sex-biased relationship with alcohol use in adulthood (drinks per month during period of greatest use). There is a robust Sex*Drinks interaction (solid-colored lines, sex interaction p < 0.05) driven by a statistically-significant negative association between volume and surface area in childhood and alcohol use in adulthood for females (red, amygdala: b = −5.2, p = 0.006/hippocampus: b = −2.6, p = 0.01), which is absent in males (blue, p’s > 0.4). This interaction holds when excluding the three males consuming > 150 drinks per month in adulthood (dashed black lines). **(B)** Center panel: surface vertex maps showing those subregions of each structure which display the statistically-significant Sex*Drinks interaction for local surface area (middle image: rostral view of amygdala and hippocampus / upper image: rostroventral view of amygdalar focus / lower image: rostrocaudal view of hippocampal focus. Neighboring scatterplots show the underlying data with age and sex residualized at each focus of interaction. All foci show a female-specific relationship between local surface area in childhood and alcohol consumption in adulthood. (For interpretation of the references to color in this figure legend, the reader is referred to the web version of this article.)

### 3.2 Sex-specific subcortical associations with peak alcohol consumption

Multivariate linear regressions revealed that the relationship between bilateral subcortical volume in youth and peak alcohol use in adulthood is significantly modulated by sex for both the amygdala and hippocampus (Sex*Drinks interaction term: amygdala: β = 4.6, SE = 1.98, p = 0.02; hippocampus: β = 7.7, SE = 3.63, p = 0.04, Table 2, Fig. 2A). These structures showed a negative association between volume in childhood and peak alcohol use in adulthood for females (amygdala: β = -5.2, SE = 1.84, p = 0.01; hippocampus: β = -8.3, SE = 3.38, p = 0.01), but not males (amygdala: β = −0.59, SE = 0.73, p = 0.43; hippocampus: β = -1.11, SE = 1.34, p = 0.41; striatum: β = 1.04, SE = 4.75, p = 0.83). Similar patterns were observed for the total surface area of each structure (Sex*Drinks interaction term: amygdala β = 3.9, SE = 1.52, p = 0.01; hippocampus β = 2.7, SE = 1.17, p = 0.02), consistent with the strong positive correlation between volume and surface area (amygdala r = 0.96, hippocampus r = 0.87). In contrast, no such associations are seen for the striatum **(**Table 2). Our findings were unaltered by exclusion of 3 males with particularly high levels of peak alcohol consumption (>150 drinks per week, z-score = 2.10), and not fully explained by co-occurring variation in total brain volume (Sex*Drinks interaction term while covarying for TBV: amygdala volume: p = 0.06, surface area: p = 0.03; hippocampus volume: p = 0.10, surface area: p = 0.05), or SES (Sex*Drinks per month co-varying for SES: amygdala volume: p = 0.04, surface area: p = 0.03; hippocampus volume: p = 0.08, surface area: p = 0.04). We further calculated volume asymmetry indices for each subcortical structure (asymmetry = ((left - right) / 0.5 * (right + left)), and then used these indices as dependent variables in models testing for interactions between sex and peak alcohol use. We did not observe statistical evidence of any such interactions (amygdala: p = 0.414, hippocampus: p = 0.697, striatum: p = 0.633), indicating that sex-biased relationships with later alcohol use do not significantly modify subcortical asymmetry (i.e. are not significantly lateralized, see **Supplementary** Table 1**)**.

**Table 2:** The Relationship between Pediatric Subcortical Volumes and Adult Alcohol Use

Variable								
	Adult Alcohol Use		Age		Sex		Sex*Drinks	
	β_1_ Coefficient (SE)	p	β_2_ Coefficient (SE)	p	β_3_ Coefficient (SE)	p	β_4_ Coefficient (SE)	p
Amygdala	−5.19 (1.84)	0.01	30.68 (12.19)	0.01	80.91 (84.01)	0.34	4.61 (1.98)	0.02
Hippocampus	−8.83 (3.38)	0.01	54.77 (22.32)	0.02	−33.75 (153.83)	0.83	7.72 (3.63)	0.04
Striatum	−5.90 (12.00)	0.62	6.20 (79.29)	0.94	899.96 (546.53)	0.10	6.94 (12.90)	0.59

*Note:* Due to the parameterization of our model, the above beta and p values for the Adult Alcohol Use term refer to the association among females.

To better resolve relationships between subcortical anatomy in childhood and alcohol use in adulthood, we conducted vertex-wise surface area analyses of all three structures. After FDR correction, surface area morphometry revealed that the global sex interactions were localized to the rostro-medial hippocampus and overlying facets of the amygdala, encompassing portions of the hippocampal subiculum and CA1 segment (Fig. 2B). The scatterplots in Fig. 2B show the data underlying the linear models, using the vertex with the most robust sex interaction in each of four structures examined as an example (i.e., left hippocampus, left amygdala, right hippocampus, right amygdala). Within the areas with a statistically significant Sex*Drinks interaction, females displayed a statistically significant negative association between pediatric morphometry and peak alcohol use while males did not (Fig. 2B). Outside of these foci, no regions of the amygdala or hippocampus were associated with peak alcohol use in either males or females. There were no striatal vertices with significant main effect or sex-modulated associations with peak drinking.

## 4 Discussion

Here we provide longitudinal evidence of sex-biased relationships between pediatric subcortical anatomy and alcohol consumption during adulthood. We provide evidence that lower amygdala and hippocampal volume and surface area during childhood is associated with greater alcohol consumption during adulthood among females but not among males. Through our longitudinal design, these results contribute to understanding of sex-specific neuroanatomical precursors for peak alcohol consumption and potential alcohol misuse in a few specific directions.

### 4.1 Subcortical anatomy and alcohol use

Our results suggest that sex-specific vulnerability to alcohol consumption is manifest years before initiation as focal variations in amygdala-hippocampal anatomy. We did not however observe any main effects associations between subcortical volume and alcohol use that were invariant between the sexes. Given that such main effects have been seen in cross-sectional studies of adults (Agartz et al., 1999, Howell et al., 2013, Makris et al., 2008, Schneider et al., 2001, Sullivan et al., 2005, Wilson et al., 2017, Wilson et al., 2015, Wrase et al., 2008), our finding of sex-modified effects may reflect the measurement of subcortical anatomy at a much younger age long before initiating alcohol use. Adult findings involve an additional phase of alcohol exposure during dynamic subcortical development (Raznahan et al., 2014) which occurs after our time of observation. Indeed, there is evidence that alcohol consumption influences subcortical anatomy among adults (Logtenberg et al., n.d., Makris et al., 2008), which may partially explain cross-sectional correlations among active or former alcohol users.

We did not observe any sex-specific associations between striatum volume and alcohol use. Relationships between striatum and alcohol use have been observed in cross-sectional research (Howell et al., 2013, Makris et al., 2008, Sullivan et al., 2005, Wrase et al., 2008), however these studies did not examine sex. It is possible that striatal anatomy might not have sex-specific associations with alcohol use. Additionally, since striatal structures develop on a distinct timeline compared to amygdala and hippocampus (Raznahan et al., 2014), sex-specific anatomical correlates of alcohol use in the striatum may not be present during childhood.

### 4.2 Sex-specific risk factors for alcohol initiation

Our finding that females showed a stronger association between subcortical volume and alcohol use than males suggests that these brain components may have a relevance for later alcohol use behaviors in females that they do not have in males. This finding is in line with previous research identifying sex differences in relationships between alcohol consumption and brain anatomy (Sawyer et al., 2017), and could relate to sex differences in the course of alcohol dependence among males and females (McHugh et al., 2018). Previous research on multiple different substances has theorized a “telescoping” progression in which females more quickly transition from initiation to disordered use and help-seeking (Piazza et al., 1989). However, this effect does not appear to generalize outside of individuals with severe substance use disorders (Keyes et al., 2010), and among the general population, males appear to more quickly transition to disordered alcohol use compared to females (Keyes et al., 2010). Thus, males may generally experience a more dramatic course of illness; however, certain females may also be uniquely prone to rapid progression of substance use disorders (McHugh et al., 2018). Our results suggest that subcortical anatomy may potentially be a predispositional factor for this rapid progression of substance use among females. This notion is supported by previous literature relating subcortical structures, particularly the basolateral amygdala (BLA), to motivational processing (Kim et al., 2017, Wassum and Izquierdo, 2015).

The BLA is thought to represent information regarding sensory-specific outcomes associated with a stimulus, and to transmit this information widely throughout the cortex (Balleine and Killcross, 2006, Janak and Tye, 2015, Wassum and Izquierdo, 2015). Animal research has identified that lesions of BLA reduce reward learning behavior in rats (Hatfield et al., 1996, Holland et al., 2002, Petrovich et al., 2005) and functional MRI in humans has found amygdala activation in response to drug related cues (Chase et al., 2011, Kühn and Gallinat, 2011). Furthermore, a large study of subcortical anatomy in alcohol use disorder has identified the BLA as a key site of anatomical sex-differences (Grace et al., 2021). Specifically, in a cross-sectional sample of adults, males with alcohol use disorder showed relative reductions in BLA volume compared to healthy control males, while this pattern was not observed in females (Grace et al., 2021). Taken together, this suggests that the amygdala, specifically the BLA, is involved in subjective valuation of sensory-relevant stimuli. Thus, speculatively, reduced amygdala volume in the BLA predicting higher alcohol use could potentially indicate that individuals with reduced BLA volume may form more favorable valuations of alcohol consumption, leading to higher subjective motivation and reinforcement. Our finding of a negative association between BLA volume and peak alcohol consumption only among females raises the possibility that BLA-dependent motivational circuits may be more relevant for initiation and/or progression of alcohol use in females than males.

The findings of localized hippocampal associations over CA1 aligns with previous research demonstrating that alcohol exposure reduces CA1 volume in rats (Livy et al., 2003, Miki et al., 2004, Murawski et al., 2012), and appears to increase microglial activation in CA1 (Boschen et al., 2016). Reduced subiculum and CA1 volume have also been recently observed in adults with alcohol use disorder compared to abstinent adults (Sawyer et al., 2020). This same study found that length of sobriety was negatively associated with CA1 volume in females but positively associated with CA1 volume in males (Sawyer et al., 2020). These reports add weight to the notion that CA1 volume is differentially associated with alcohol consumption between males and females - namely, with reduced CA1 volume being more closely associated with increased drinking behavior in females than in males.

### 4.3 Limitations and future directions

Our findings should be considered in light of several study limitations and caveats. First, none of the participants in our study had a diagnosed psychiatric disorder at enrollment in childhood. This limits the generalizability of our findings to instances where alcohol use emerges on a background of pediatric psychopathology but increases interpretability of our findings by limiting the possibility of confounding influences from pre-existing mental health issues prior to alcohol exposure. Second, our study has a modest sample size relative to most longitudinal neuroimaging datasets, but a relatively large sample size for the unusually long period of follow-up. This fundamental tradeoff between study longevity and sample size will be better addressed by ongoing multi-center neuroimaging studies of brain development, such as the ABCD study (Bjork et al., 2017). The ABCD cohort will be well situated to test the reproducibility of our findings and connect them to longitudinal behavioral covariates once sufficient follow-up time has passed. Third, while we did not observe an effect of age at scanning, scans were performed during a wide developmental window during a period of dynamic neurodevelopment. Future studies will be able to resolve the relationship between developmental trajectories and adult alcohol use by conducting longitudinal measurements of brain anatomy. Longitudinal analyses will be particularly important for studying sex-differences given the likelihood of spatiotemporal sex-differences in brain development. Fourth, for some participants, the age at follow-up falls before the typical developmental window of peak alcohol intake, or before participants have reached the age to legally consume alcohol meaning that our recorded levels of peak drinking may be underestimated for some study participants. However, countering this concern is the fact that very few participants report that their period of peak drinking is at the time of follow-up - suggesting that most participants were seen with lower current drinking than a prior peak. Fifth, we did not measure SES during adulthood, which may have an effect on peak alcohol consumption. Finally, it is crucial to note that sex and gender may have different relationships with alcohol usage, but our study only considers the biological variable of sex. Future research must consider how the numerous social and environmental factors associated with gender expression might act in concert with biological sex to give rise to disordered alcohol use.

## 5 Summary

Our study presents evidence of sex-specific associations between pediatric subcortical anatomy and alcohol use in adulthood. Specifically, using anatomy as a marker, our study suggests that subcortical systems in childhood may show a distinct relevance for later alcohol use in females which is not apparent in males. These findings propose a potential contributing factor to well-documented sex-differences in initiation and progression of alcohol use.

## 6 Funding and disclosures

This work was supported by the National Institute of Mental Health Intramural Research Program (Clinical trial reg. No. NCT00001246, clinicaltrials.gov; NIH Annual Report Number, ZIAMH002794). The authors have no conflicts of interest to disclose.
